# Combined Bisoprolol and Megestrol Acetate Improves Survival and Preserves Cardiac Performance in a Rat Model of Cancer Cachexia

**DOI:** 10.1002/jcsm.70313

**Published:** 2026-05-22

**Authors:** Zihao Chen, Siqi Wang, Sandra Palus, Stefan D. Anker, Wolfram Doehner, Jochen Springer

**Affiliations:** ^1^ Berlin Institute of Health Center for Regenerative Therapies (BCRT) Charité – Universitaetsmedizin Berlin Berlin Germany; ^2^ Department of Cardiology (CVK) Charité – Universitaetsmedizin Berlin Berlin Germany; ^3^ German Centre for Cardiovascular Research (DZHK) Partner Site Berlin Berlin Germany

**Keywords:** body composition, body weight, cancer cachexia, cardiac function, heart, survival

## Abstract

**Background:**

Cancer cachexia is associated with rapid body wasting and cardiac dysfunction. Bisoprolol (BIS) and megestrol acetate (MA) improve selected cachexia phenotypes in the Yoshida AH‐130 model, but their combined effects are not defined. We tested reduced‐dose combined treatment of BIS and MA (COMB) on survival and organ‐specific outcomes.

**Methods:**

Male Wistar Han rats (approx. 200 g) received intraperitoneal Yoshida AH‐130 hepatoma cells and were treated with placebo (PL, *n* = 49), BIS (5 mg/kg/day, *n* = 23), MA (100 mg/kg/day, *n* = 10) or COMB (BIS 3.75 mg/kg/day + MA 75 mg/kg/day, *n* = 16). Outcomes included survival, echocardiography (baseline and Day 11), body composition (EchoMRI), terminal tissue weights, food intake and spontaneous locomotor activity.

**Results:**

COMB reduced mortality versus placebo (HR = 0.21, *p* < 0.0001) and had the lowest HR among treatment groups. BIS also reduced mortality versus PL (HR = 0.32, *p* = 0.0007), whereas MA did not reach statistical significance (HR = 0.49, *p* = 0.082). COMB did not differ significantly from BIS (HR for COMB vs. BIS = 0.48, *p* = 0.28) or MA (HR for COMB vs. MA = 0.23, *p* = 0.06). On Day 11, COMB showed higher LVEF than PL (64.1% ± 9.7% vs. 50.5% ± 12.8%, *p* < 0.01), higher LV stroke volume (186 ± 49 μL vs. 112 ± 55 μL, *p* < 0.001) and higher LV mass (515 ± 95 mg vs. 429 ± 65 mg, *p* < 0.01); changes from baseline were smaller in COMB than PL for LVEF (Δ −12.0% ± 8.9% vs. −24.3% ± 16.2%, *p* < 0.05) and LV mass (Δ −57 ± 93 mg vs. −137 ± 79 mg, *p* < 0.05). Body weight, fat mass and lean mass decreased in all groups; COMB showed smaller reductions than PL (*p* < 0.05) but did not differ from BIS or MA (*p* > 0.05). BAT weight was higher in COMB than PL (*p* < 0.001) and higher than BIS (*p* < 0.001). Food intake on Day 11 was higher than PL in all active groups, and locomotor activity was higher than PL in MA and COMB.

**Conclusions:**

COMB reduced mortality versus placebo and showed the lowest HR among treatment groups, accompanied by preserved cardiac performance and a distinct BAT response. Differences versus monotherapy were not statistically significant. Future studies should test optimized dosing and include exposure assessment and tissue profiling to determine benefit over monotherapy and to clarify the basis of the cardiac and BAT phenotypes.

## 1 Introduction

Cancer cachexia is a multifactorial syndrome characterized by progressive weight loss with depletion of skeletal muscle and adipose tissue that cannot be fully reversed by nutritional support. It is common in advanced cancer and is associated with reduced treatment tolerance, impaired physical function and increased mortality [1, 2, 3].

Cachexia is increasingly viewed as a systemic, multi‐organ disorder and cardiac wasting has emerged as a relevant component in both clinical cohorts and experimental models and is linked to adverse outcomes [4, 5]. The Yoshida AH‐130 hepatoma model shows rapid cachexia progression with early cardiac involvement, enabling controlled testing of interventions targeting cardiac outcomes [6].

Current treatment options for cancer cachexia remain limited. Guideline‐recommended strategies, including appetite stimulants and single‐pathway metabolic modulators, often yield modest benefit and effective multi‐target approaches are still lacking [7, 8]. Combination regimens addressing complementary pathways may be required to influence both systemic wasting and organ‐specific dysfunction [9, 10].

Bisoprolol (BIS), a β_1_‐selective adrenergic receptor blocker, has shown survival benefit and cardioprotective effects in preclinical cachexia models, including preservation of cardiac mass and improvements in functional indices [6, 11]. In parallel, megestrol acetate (MA) is widely used as an orexigenic agent to improve appetite and weight and has been linked to changes in inflammatory and proteolysis/autophagy‐associated signalling, including in cardiac tissue [12, 13]. These agents represent a rational pair to test a combined strategy targeting both cardiac and systemic domains.

Despite these data, the combined effects of BIS and MA on survival and organ‐specific outcomes in experimental cancer cachexia have not been systematically evaluated. We therefore tested BIS and MA as monotherapies and in combination (COMB; reduced‐dose combination) in the AH‐130 rat model and assessed survival, echocardiographic cardiac function, body composition, tissue weights, food intake and spontaneous activity. We hypothesized that co‐treatment may provide broader phenotypic effects than either agent alone.

## 2 Methods

### 2.1 Animals and Tumour Inoculation

The cancer cachexia animal model was established through intraperitoneal injection of Yoshida Hepatoma AH‐130 cells into male Wistar Han rats (weighing approximately 200 g; Charles River, Sulzfeld, Germany), following previously described protocols [6, 14]. Animals were housed under specific pathogen‐free conditions at the Centre for Cardiovascular Research in Berlin, Germany, with ambient temperature maintained at 22°C and a 12‐h light–dark cycle. Food and water were provided ad libitum. Rats were randomly assigned to either a placebo group (PL, *n* = 49) or treatment groups. The treatment cohort was further subdivided into a BIS monotherapy group (5 mg/kg/day, *n* = 23), a MA monotherapy group (100 mg/kg/day, *n* = 10) and a combination therapy group (COMB, 3.75 mg/kg/day bisoprolol + 75 mg/kg/day MA, *n* = 16). The doses in the combination group corresponded to 75% of the effective monotherapy doses for each agent.

### 2.2 Food Intake and Spontaneous Activity

Quality‐of‐life–related measures were assessed through measurements of spontaneous activity and food intake, which were recorded over 24‐h periods both 1 day prior to tumour inoculation and on Day 11 using previously described methodologies [6, 15]. Animals were individually housed and provided with 100 g of food. Spontaneous locomotor activity was monitored continuously for 24 h using an infrared sensor–based system (Supermex Locomotor System; Muromachi Kikai Co Ltd., Tokyo, Japan).

### 2.3 Body Composition

Whole‐body fat mass and lean mass were analysed using quantitative nuclear magnetic resonance (NMR) spectroscopy (EchoMRI‐700, Echo Medical Systems, Houston, Texas, USA) 1 day prior to tumour inoculation and again on the day of euthanasia, in accordance with established protocols. The EchoMRI system has a reported sensitivity of 2 g and separates fat and lean compartments based on differences in NMR relaxation properties of hydrogen nuclei (static magnetic field 0.05 T; radiofrequency pulses at 2 MHz) [16]. During measurement, each rat was measured awake and briefly restrained in a dedicated tube to minimize movement for approximately 90 s. EchoMRI reports whole‐body fat and lean mass and was not used to derive organ or tissue‐specific volumes. Skeletal muscle outcomes were assessed by terminal wet weights.

### 2.4 Tissue Collection and Terminal Tissue Weights

At the end of the intervention period, rats were euthanized and tissues were rapidly excised and weighed. Skeletal muscle weights were assessed by wet weights of gastrocnemius and tibialis anterior. Adipose depots (epididymal fat and brown adipose tissue) and heart were also collected and weighed.

### 2.5 Cardiac Function

Echocardiography was performed using a high‐resolution Vevo 770 system (VisualSonics, Toronto, Canada) at baseline and on Day 11 after tumour inoculation, following previously described methods [17]. Briefly, rats were anaesthetized with 1.5% isoflurane, placed in a supine position on a temperature‐controlled platform to maintain physiological body temperature, and the left thoracic region was shaved to facilitate acoustic access. Cardiac function and structural dimensions were assessed using both B‐mode and M‐mode imaging.

### 2.6 Statistical Analysis

Results are presented as mean ± SEM and were analysed using GraphPad Prism Version 9.0 (GraphPad Software Inc., La Jolla, California, USA). Normality of data distribution was assessed using the Kolmogorov–Smirnov test. For normally distributed data, group comparisons were performed using one‐way ANOVA followed by Tukey's post hoc test; non‐normally distributed data were analysed using the Kruskal–Wallis test followed by Dunn's post hoc test. Survival analysis was conducted using Kaplan–Meier curves and log‐rank test, with additional assessment via Cox proportional hazards regression. A *p*‐value less than 0.05 was considered statistically significant.

## 3 Results

### 3.1 Survival

**FIGURE 1:**
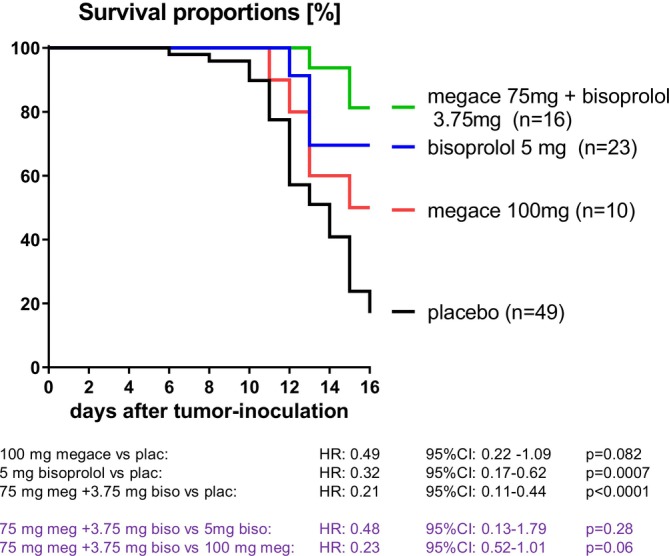
Kaplan–Meier survival curves. Survival of tumour‐bearing rats treated with placebo (*n* = 49), BIS (bisoprolol 5 mg/kg/day, *n* = 23), MA (MegaceES 100 mg/kg/day, *n* = 10) or COMB (bisoprolol 3.75 mg/kg/day + MegaceES 75 mg/kg/day, *n* = 16). Hazard ratios (HR) with 95% confidence intervals (CI) and *p*‐values for the indicated comparisons are shown in the figure.

Kaplan–Meier analysis showed reduced mortality in the combination group versus placebo (HR = 0.21, *p* < 0.0001; Figure 1). BIS also reduced mortality versus placebo (HR = 0.32, *p* = 0.0007), whereas MA did not reach statistical significance (HR = 0.49, *p* = 0.082). The combination group did not differ significantly from BIS (HR for COMB vs. BIS = 0.48, *p* = 0.28) or MA (HR for COMB vs. MA = 0.23, *p* = 0.06).

### 3.2 Cardiac Function and Structure

At baseline, left ventricular mass (LVM) was lower in BIS than placebo (*p* < 0.05), whereas other baseline echocardiographic parameters did not differ among groups. On Day 11 of treatment, COMB showed a smaller decline in left ventricular ejection fraction (LVEF) than placebo (ΔLVEF −12.0% ± 8.9% vs. −24.3% ± 16.2%, *p* < 0.05) and higher LVEF at Day 11 (64.1% ± 9.7% vs. 50.5% ± 12.8%, *p* < 0.01). COMB showed a smaller loss of LVM than placebo (ΔLVM −57 ± 93 mg vs. −137 ± 79 mg, *p* < 0.05) and higher LVM at day 11 (515 ± 95 mg vs. 429 ± 65 mg, *p* < 0.01). Left ventricular stroke volume (LVSV) at Day 11 was higher in COMB than placebo (186 ± 49 μL vs. 112 ± 55 μL, *p* < 0.001). In MA, ΔLVEF was −3.2% ± 24.9% (*p* < 0.01 vs. placebo). In BIS, ΔLVM was −5 ± 112 mg (*p* < 0.001 vs. placebo) and LVSV at Day 11 was 160 ± 82 μL (*p* < 0.05 vs. placebo). Changes in left ventricular end‐diastolic volume (LVEDV) and left ventricular fractional shortening (LVFS) did not differ significantly among groups (Table 1). Individual‐animal echocardiography data are shown in Figure S1.

**TABLE 1:** Echocardiographic parameters at baseline and Day 11 after tumour inoculation.

Parameter	Unit	Placebo	5 mg/kg/d bisoprolol	100 mg/kg/d MegaceES	3.75 mg/kg/d biso + 75 mg/kg/d Megace
LVEF baseline	%	75.5 ± 8.1	74.7 ± 8.6	72.5 ± 11.5	76.1 ± 7.5
LVEF Day 11	%	50.5 ± 12.8	59.5 ± 12.3	67.2 ± 17.5**	64.1 ± 9.7**
Delta LVEF	%	−24.3 ± 16.2	−20.8 ± 15.1	−3.2 ± 24.9**	−12.0 ± 8.9*
LVFS baseline	%	51 ± 8	48 ± 7	53 ± 8	53 ± 5
LVFS Day 11	%	32 ± 10	36 ± 9	45 ± 15	37 ± 6
Delta LVFS	%	−19 ± 13	−15 ± 13	−8 ± 15	−15 ± 4
LVEDV baseline	μL	255 ± 39	266 ± 46	219 ± 45*	262 ± 31
LVEDV Day 11	μL	186 ± 61	260 ± 83	188 ± 61	286 ± 42
Delta LVEDV	μL	−64 ± 72	−10 ± 114	−27 ± 50	24 ± 61
LVESV baseline	μL	60 ± 22	67 ± 24	61 ± 21	64 ± 28
LVESV Day 11	μL	88 ± 26	103 ± 52	71 ± 26	100 ± 22
Delta LVESV	μL	21 ± 31	42 ± 40	6 ± 26	36 ± 29
LVSV baseline	μL	198 ± 34	199 ± 43	162 ± 50*	198 ± 19
LVSV Day 11	μL	112 ± 55	160 ± 82*	135 ± 65	186 ± 49***
Delta LVSV	μL	−85 ± 67	−52 ± 89	−10 ± 56	−11 ± 54*
LVM baseline	mg	549 ± 112	484 ± 56*	582 ± 94	590 ± 79
LVM Day 11	mg	429 ± 65	469 ± 79	482 ± 99	515 ± 95**
Delta LVmass	mg	−137 ± 79	−5 ± 112***	−133 ± 124	−57 ± 93*

*Note:* Values are mean ± SEM. Baseline indicates measurements prior to tumour inoculation; Day 11 indicates measurements on Day 11 after tumour inoculation; Δ indicates change from baseline to Day 11.

Abbreviations: LVEDV, left ventricular end‐diastolic volume; LVEF, left ventricular ejection fraction; LVESV, left ventricular end‐systolic volume; LVFS, left ventricular fractional shortening; LVM, left ventricular mass; LVSV, left ventricular stroke volume.

* *p* < 0.05.

** *p* < 0.01.

*** *p* < 0.001 versus placebo at the same time point. Statistical tests are described in Section 2.

### 3.3 Body Weight and Body Composition

**FIGURE 2:**
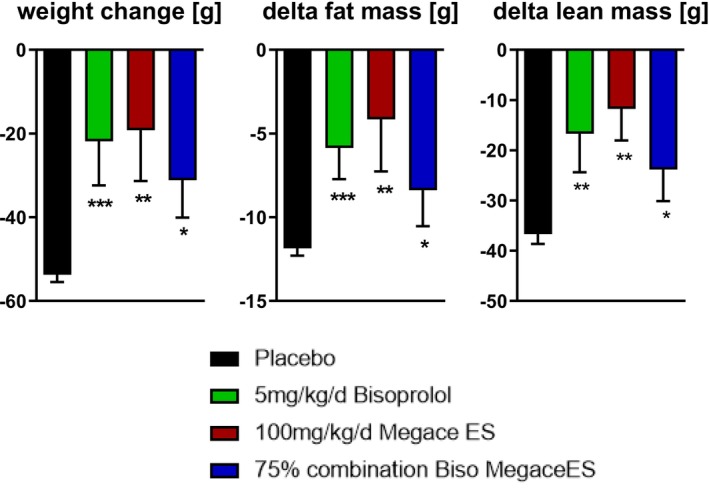
Changes in body weight and body composition. Change in total body weight, fat mass and lean mass from baseline to the end of the intervention period in placebo, BIS, MA and COMB groups. Data are mean ± SEM. *p < 0.05, **p < 0.01, ***p < 0.001 versus placebo.

At the end of the intervention period, body weight, fat mass and lean mass decreased in all groups. Compared with placebo, COMB showed smaller reductions in body weight, fat mass and lean mass (Figure 2). The mean changes in COMB were intermediate between placebo and the monotherapy groups (Figure 2).

### 3.4 Terminal Tissue Weights

**FIGURE 3:**
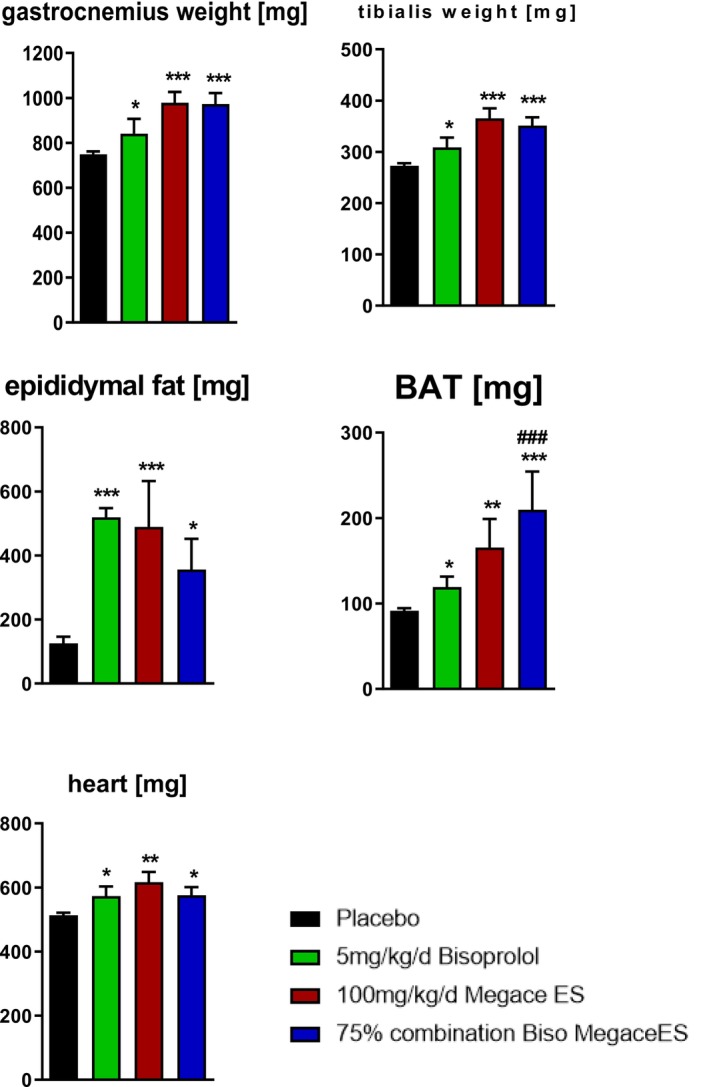
Terminal tissue weights. Weights of gastrocnemius, tibialis anterior, epididymal fat, brown adipose tissue (BAT) and heart at the end of the intervention period in placebo, BIS, MA and COMB groups. Data are mean ± SEM. *p < 0.05, **p < 0.01, ***p < 0.001 versus placebo. ###*p* < 0.001 versus BIS.

At the end of the intervention period, COMB showed higher weights of gastrocnemius, tibialis anterior, epididymal fat, BAT and heart than placebo (*p* < 0.05 for each). BAT weight in COMB also exceeded BIS (*p* < 0.001 vs. BIS). BIS and MA differed from placebo in the same direction across these tissues (*p* < 0.05 for each) (Figure 3).

### 3.5 Food Intake and Spontaneous Activity

**FIGURE 4:**
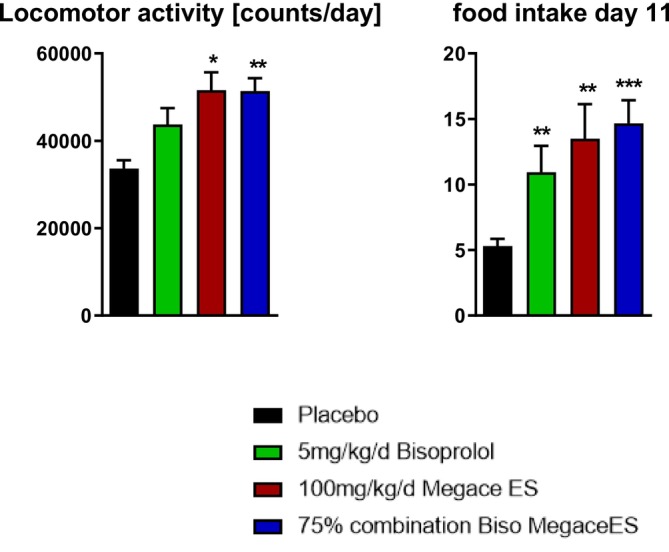
Food intake and locomotor activity. Food intake on Day 11 and locomotor activity (counts/day) in placebo, BIS, MA and COMB groups. Data are mean ± SEM. *p < 0.05, **p < 0.01, ***p < 0.001 versus placebo.

Food intake on Day 11 was higher in BIS, MA and COMB than in placebo (Figure 4). Locomotor activity was higher in MA and COMB than in placebo, whereas BIS did not differ significantly from placebo (Figure 4).

## 4 Discussion

This study tested whether COMB changes key cachexia phenotypes in the Yoshida AH‐130 model. COMB showed the lowest hazard ratio for mortality versus placebo among the treatment groups (HR = 0.21; Figure 1). COMB reduced mortality risk versus placebo and showed a broad pattern of preserved cardiac structure and function. In contrast, COMB did not differ significantly from either monotherapy for survival, and whole‐body lean and fat mass were not improved beyond monotherapy. A distinct tissue finding was BAT, which was higher in COMB than BIS.

Cancer cachexia affects multiple organs, and cardiac wasting is increasingly recognized as clinically relevant. Clinical observations report loss of LVM in advanced cancer and associations with functional impairment and mortality, even when ejection fraction is preserved, consistent with reduced cardiac reserve [18]. The AH‐130 model shows rapid cardiac atrophy and functional deterioration, providing a suitable platform to assess interventions targeting cardiac outcomes in severe cachexia [19, 20]. Echocardiography and behavioural readouts were assessed on Day 11, a standardized time point in the AH‐130 model when cachexia‐related cardiac deterioration becomes evident and cardiac function has been linked to outcome [6].

Against this background, our data extend previous work on BIS and MA by assessing their co‐administration. Across echocardiographic readouts, monotherapies showed different patterns, whereas COMB showed concurrent preservation of several cardiac indices versus placebo. This pattern likely reflects complementary physiological effects of BIS and MA. However, because COMB did not differ significantly from BIS or MA for survival, the present dataset does not provide formal evidence for benefit over monotherapy or interaction effects. Any interaction interpretation should therefore be treated as hypothesis‐generating and requires adequately powered designs with pre‐specified interaction testing.

One possible explanation for the lowest hazard ratio with COMB versus placebo is the concurrent pattern of cardiac and functional effects observed with BIS and MA, respectively. In COMB, these effects occurred in parallel, which may help maintain cardiac reserve during rapid disease progression. This interpretation remains cautious because COMB did not differ significantly from either monotherapy for survival, and drug exposure was not measured.

We used BIS because it is a β_1_‐selective antagonist with reproducible efficacy on survival and cardiac outcomes in AH‐130 cachexia in our prior dose‐finding work [6]. Importantly, beta‐blockers do not exert uniform class effects in experimental cachexia. Atypical compounds such as S‐pindolol or ACM‐001 have additional pharmacological properties (e.g., β_2_‐intrinsic sympathomimetic activity and central effects) that can directly influence appetite, activity and tissue metabolism [21, 22]. These differences may lead to different interaction profiles when combined with MA and should be addressed in future comparative studies.

Mechanistic interpretation is limited in the current cohort because no molecular or histological endpoints were measured. Based on previous studies, β_1_‐adrenergic blockade has been linked to reduced sympathetic drive and modulation of proteostasis‐related pathways, whereas MA primarily acts as an orexigenic agent and has been associated with changes in inflammatory and autophagy‐related signalling in the myocardium [6, 12]. The observed pattern may reflect partially non‐overlapping processes, but this remains untested without direct measurements.

A notable finding was the dissociation between cardiac outcomes and whole‐body composition. Despite favourable cardiac indices and lower mortality risk versus placebo, COMB did not enhance preservation of lean and fat mass beyond monotherapy. Several explanations are possible. First, improved survival may extend exposure to a catabolic milieu and thereby reveal ongoing peripheral wasting over time. Second, the reduced‐dose regimen (75% of each effective monotherapy dose) may shift systemic versus organ‐specific effects. Third, cachexia involves tissue‐specific metabolic reprogramming; improved cardiac performance does not necessarily translate into whole‐body composition preservation within the same time window. These points support follow‐up studies focused on dose optimization and longitudinal tissue trajectories.

An interesting pattern emerged across adipose depots. Epididymal fat (a representative WAT depot) increased versus placebo across active treatments, but it did not show the same between‐treatment separation as BAT. In contrast, BAT weight was highest in COMB and exceeded BIS. BAT mass is closely linked to thermogenic activation and depletion of lipid stores, whereas WAT wet weight reflects a broader balance of lipid mobilization and deposition. BIS may dampen sympathetic and adrenergic activation of BAT, whereas MA increases food intake and favours fat deposition, which together could facilitate preservation of BAT mass [23, 24, 25, 26]. Importantly, we assessed depot weights rather than thermogenic or lipolysis markers; increased BAT weight could reflect restored lipid stores or BAT ‘whitening’. Future studies should include BAT and WAT histology and thermogenic markers (e.g., UCP1 and mitochondrial content) across multiple WAT depots to clarify functional relevance.

Locomotor activity was higher than placebo in MA and COMB on Day 11, whereas BIS did not differ from placebo. The activity response in COMB was similar to MA, consistent with the activity benefit being associated with MA‐containing regimens. Food intake on Day 11 was higher than placebo in all active treatment groups. These endpoints provide functional context, but the present data do not allow conclusions about mediation between intake and activity changes and survival.

Several limitations should be acknowledged. The study was not powered to quantify drug interaction effects, and COMB did not differ significantly from each monotherapy. Drug exposure was not measured. Pharmacokinetics can change in cachexia due to altered hepatic metabolism and renal elimination, which may contribute to variability and complicate interpretation [27]. Mechanistic inference is limited by the absence of molecular and histological analyses. Baseline differences in selected measures (e.g., LVM) further highlight the need for adequately powered randomization and pre‐specified analyses in future studies.

In summary, COMB was associated with improved survival versus placebo and a broad pattern of preserved cardiac performance, with a distinct BAT response, whereas whole‐body composition effects were not enhanced beyond monotherapy. Larger cohorts with exposure assessment, dose optimization, and mechanistic profiling are needed to define the relative benefit over monotherapy and to clarify the basis of the observed phenotypic pattern.

## Funding

The authors have nothing to report.

## Conflicts of Interest

The authors declare no conflicts of interest.

## Supporting information


**Figure S1:** Individual‐animal echocardiography data. Individual values (dots) and group means (horizontal bars) for left ventricular ejection fraction (LVEF), left ventricular fractional shortening (LVFS), left ventricular stroke volume (LVSV), and left ventricular mass (LVM) at baseline, Day 11, and change from baseline (Δ) in placebo, BIS, MA, and COMB groups. Δ indicates change from baseline to Day 11. Statistical comparisons are reported in Table 1; Figure S1 is provided to display individual‐animal distributions.
