# Supplementary figures and images for: Combined Bisoprolol and Megestrol Acetate Improves Survival and Preserves Cardiac Performance in a Rat Model of Cancer Cachexia
Source: J Cachexia Sarcopenia Muscle. 2026 May 22;17(3):e70313. doi: 10.1002/jcsm.70313 (PMC13240587; doi:10.1002/jcsm.70313)

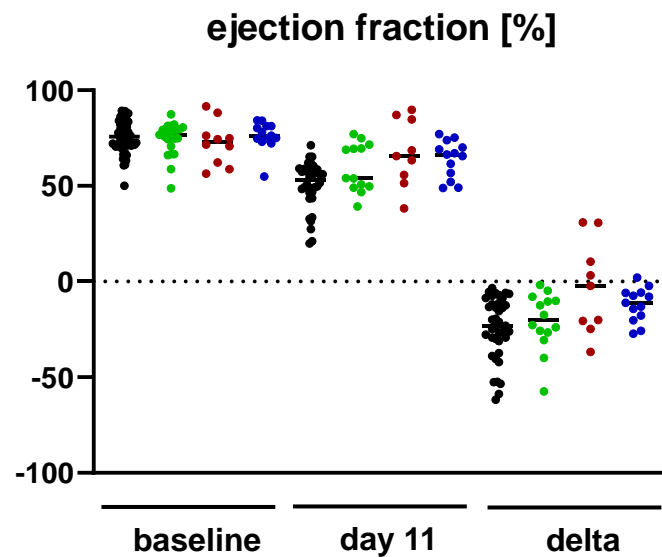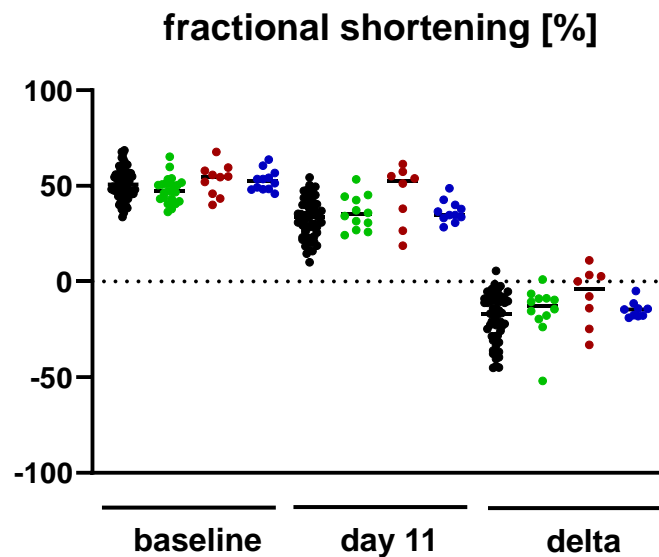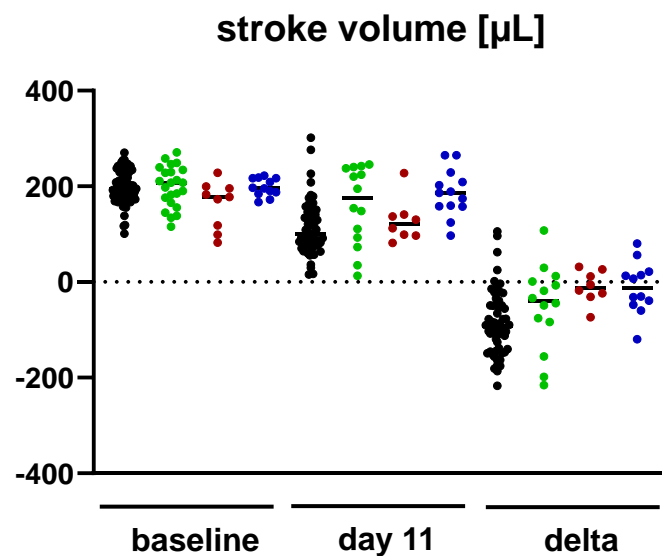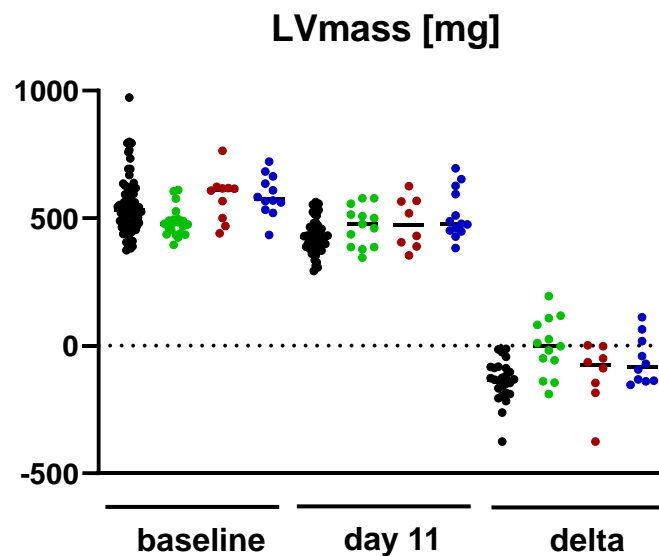

- placebo
- 5mg/kg/d Bisoprolol
- 100mg/kg/d Megace ES
- 75% combination Biso MegaceES

Supplement: Supplementary file 1 — Figure S1: Individual‐animal echocardiography data. Individual values (dots) and group means (horizontal bars) for left ventricular ejection fraction (LVEF), left ventricular fractional shortening (LVFS), left ventricular stroke volume (LVSV), and left ventricular mass (LVM) at baseline, Day 11, and change from baseline (Δ) in placebo, BIS, MA, and COMB groups. Δ indicates change from baseline to Day 11. Statistical comparisons are reported in Table 1; Figure S1 is provided to display individual‐animal distributions. [file JCSM-17-e70313-s001.pdf]
